# Supplementary material for: Symmetry breaking propulsion of magnetic microspheres in nonlinearly viscoelastic fluids
Source: Nat Commun. 2021 Feb 18;12:1116. doi: 10.1038/s41467-021-21322-0 (PMC7893017; doi:10.1038/s41467-021-21322-0)
Supplement: Supplementary file 1 — Supplementary Information [file 41467_2021_21322_MOESM1_ESM.pdf]

Supporting Information: Symmetry Breaking Propulsion of Magnetic Microspheres in Nonlinearly Viscoelastic Fluids

Louis William Rogowski[1], Jamel Ali[2], Xiao Zhang[1], James N. Wilking[3], Henry C. Fu[4*], Min Jun Kim[1*]

[1]Department of Mechanical Engineering, Southern Methodist University, Dallas, TX 75205, U.S.A.
[2]Department of Chemical and Biomedical Engineering, FAMU-FSU College of Engineering, Tallahassee, FL 32310, U.S.A.
[3]Department of Chemical and Biological Engineering, Montana State University, Bozeman, MT, 59717, U.S.A.
[4]Department of Mechanical Engineering, The University of Utah, Salt Lake City, UT 84112, U.S.A.
Emails: henry.fu@utah.edu and mjkim@lyle.smu.edu

Supplementary Note 1: Propulsion and Transverse Velocities

In this manuscript we observed the propulsion of microparticles through synthetic mucus. As defined in the main text, the propulsion direction is parallel to the rotation axis of the magnetic field. Microparticles were observed to move in both the propulsion direction and the transverse direction perpendicular to the rotation axis. The transverse velocity was influenced by proximity to the boundaries of the sample chamber (2 mm in diameter × 1 mm in height) and was proportional to the rotational frequency of the microparticle. For simplicity, transverse velocity can be thought

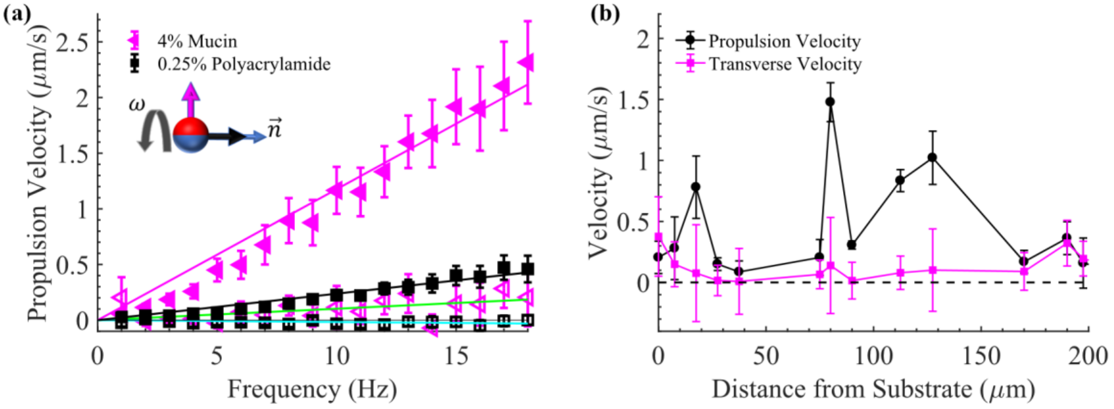

Supplementary Fig. 1. Propulsion of microparticles in a sample chamber. (a) Propulsion velocity and transverse velocity vs. rotational frequency in 4% mucin and 0.25% polyacrylamide. The inset of (a) shows a microparticle (red and blue hemispheres represent dipoles) rotated under a constant frequency (ω) along a heading direction (**n**); black and magenta arrows represent propulsion direction and transverse direction, respectively. Filled symbols represent propulsion velocity while open symbols represent transverse velocity for each fluid. The coefficient of determination (r^2) for the propulsion and transverse fittings (magenta and green solid lines) of 4% mucin were 0.965 and 0.24; the propulsion and transverse velocity fittings of 0.25% polyacrylamide (black and cyan solid lines) were 0.945 and -0.3417. Microparticles were rotated counterclockwise when viewed from behind the heading vector. (b) Propulsion and transverse velocity vs. distance from the substrate in 4% mucin. Transverse propulsion was significantly increased when the microparticle was in contact with the boundary of the sample chamber; however, transverse propulsion is present at all distances but highly variable at larger distances. Random variations in propulsion and transverse velocity at larger distances are likely due to heterogeneities within the mucus hindering microparticle translation. Data are presented as mean values +/- standard error for (a) and +/- standard deviation for (b). Six independent particles were examined in (a) for 4% mucin and seven were examined in 0.25% polyacrylamide; all particles had at least three independent trials each. One particle was tracked at each depth in (b) over a single experiment, with the standard deviation representing the instantaneous propulsion velocity over the course of the experiment. Source data are provided as a source data file.

of as the microparticle rolling like a wheel on a nearby surface as it rotates. Supplementary Figure 1 (a) shows the propulsion and transverse velocities of 10 μm diameter microparticles for different frequencies in both fluids, with the inset of (a) showing the propulsion and transverse directions. While the transverse effect could become significant in the mucin solution, its effect was negligible in the 0.25% polyacrylamide solution. All particles examined were greater than 100 μm from the boundaries of the sample chamber and at least six particles were averaged together per fluid with at least 3 trials each. Twenty microparticles had their propulsion and transverse velocities analyzed at different distances away from the bottom of the chamber in 4% mucin [Supplementary Figure 1 (b)]. A sharp increase in transverse velocity can be seen when the microparticles were in contact with the bottom of the chamber. Transverse velocity was significantly reduced when the microparticle was in the bulk fluid medium greater than 20 μm from the boundary. Due to the heterogeneous viscoelastic nature of mucus and mucin glycoprotein entanglements, this rolling effect can be experienced by microparticles even if they are far from the surface of the sample chamber, as localized regions of mucus can act as deformable surfaces. The velocity along the transverse direction was considerably less than the propulsion velocities, especially when far from the boundaries of the chamber. Distance from the substrate did not appear to systematically affect the propulsion velocity of individual microparticles, instead we attribute variations in the propulsion velocity to heterogeneities within the mucus. For rolling motion close to the boundary, it is expected that the transverse velocity should increase linearly with frequency. We therefore tested the frequency dependence of transverse velocity for particles near the boundary (~1 μm). The results are shown in Supplementary Figure 2 which compares the transverse velocities of microparticles in both 4% mucin and 0.25% polyacrylamide; each fluid had at least 5 particles averaged together, each with at least 3 trials. Unlike the results shown previously for transverse velocities in bulk fluid [Supplementary Figure 1 (a)], microparticles near the surface experienced a linear increase in transverse velocity as frequency increased and at much higher magnitudes. We note that for these microparticles very near the boundary, we generally observed much smaller propulsion velocities than in bulk fluid, but we did not further explore how boundary effects influence propulsion velocities.

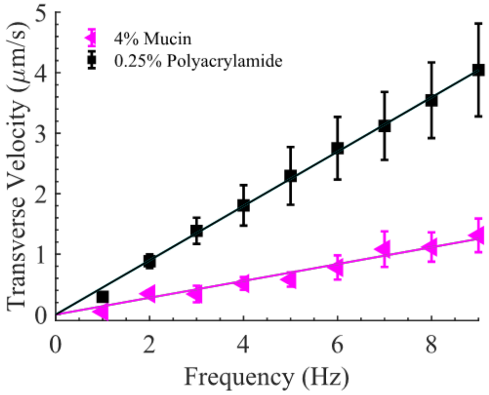

Supplementary Fig. 2. Transverse velocity vs. frequency for microparticles close to the substrate of the sample chamber. The r^2 values were 0.9634 and 0.9973 for 4% mucin and 0.25% polyacrylamide, respectively. Frequency range was reduced from other experiments to prevent microparticles from rolling out of the field of view. Data are presented as mean values +/- standard error. Seven independent particles were examined in 4% mucin and 5 were examined in 0.25% polyacrylamide; all particles had at least three trials each. Source data are provided as a source data file.

Supplementary Note 2: Static Magnetic Field Manipulation of Microparticles

As discussed in detail in the main text, the application of a static magnetic field changes the propulsion direction of the microparticles, however, the static field's effect on transverse velocity was not significant. Supplementary Figure 3 (a) shows a sample experiment of a microparticle rotated at 15 Hz while the static magnetic field increased from -5 to 5 mT in 1 mT increments, during which it initially propelled along the negative *x*-axis and then along the positive *x*-axis after the static field became positive. The transverse velocity caused the microparticle in this experiment to gradually move in the positive *y*-direction and was not affected by the change in static magnetic field. These results are quantified in Supplementary Figure 3 (b), which shows the propulsion velocity, and Supplementary Figure 3 (c), which shows the transverse velocity as functions of

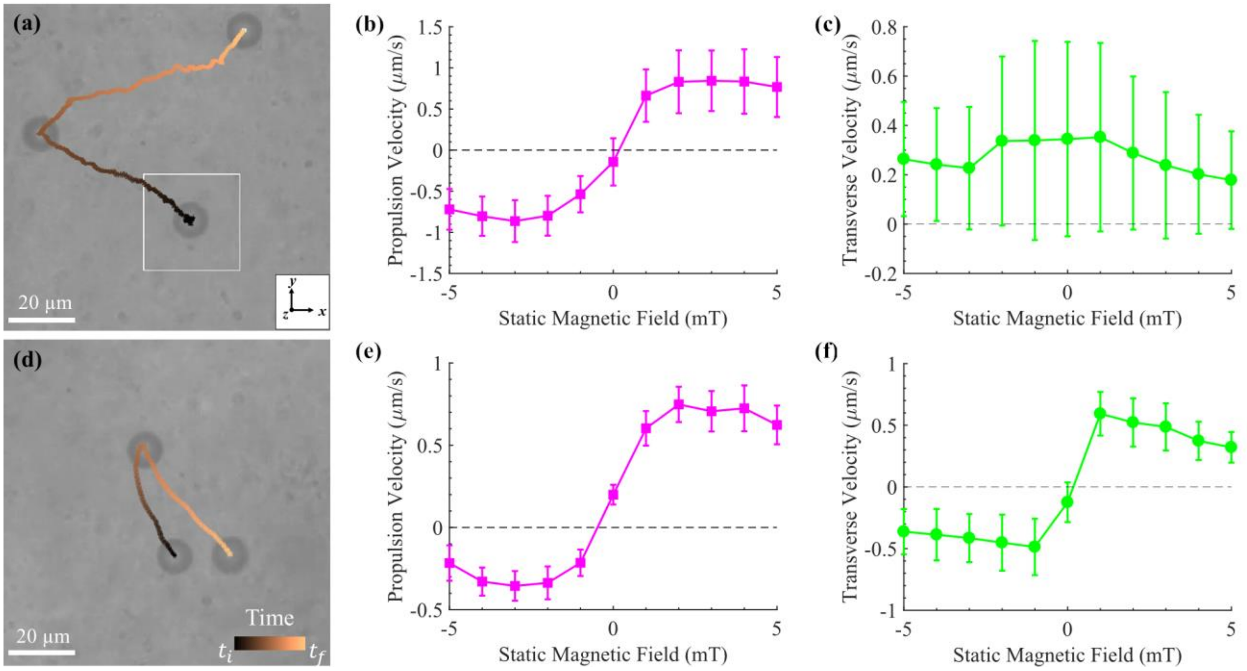

Supplementary Fig. 3. Combined effect of static field and rotation direction on velocity of 10 µm diameter microparticles. (a) Static magnetic field increased from -5 to 5 mT at 1 mT increments along the heading direction of 0°. (b) The propulsion velocity for microparticles and (c) the transverse velocity for microparticles during experiments like the one shown in (a). While the propulsion velocity is directly related to the static magnetic field, the transverse velocity was unaffected. (d) Same experiment as (a), but at 0 mT the rotation of the magnetic field was switched from counterclockwise to clockwise from behind the heading vector of 0° in the *x*-*y* plane. (e) The propulsion velocity and (f) the transverse velocities for the experiments like the one shown in (d). In this situation, both propulsion and transverse velocities are now affected, with the propulsion velocity only affected by the static magnetic field, while the transverse velocity is affected by the change in rotation direction of the rotating magnetic field. The dashed lines in (b), (c), (e), and (f) represent a propulsion velocity of 0 µm/s. Total time of experiments in (a) and (d) were 67 seconds and 46 seconds, respectively. Data are presented as mean values +/- standard error. Four microparticles were examined in (b, c, e, and f) and averaged together; all microparticles had at least three trials each. Source data are provided as a source data file.

static field; these graphs show the averaged results of four microparticles with each tested under at least three trials. As the static field increases, the average propulsion velocity changes direction at 0 mT [Supplementary Figure 3 (b)] but the transverse velocity remains the same. When the rotating magnetic field was switched from a counterclockwise direction to clockwise from behind the heading vector at 0 mT [an example experiment can be seen in Supplementary Figure 3 (d)], it was found that the propulsion velocity in Supplementary Figure 3 (e) remains similar to Supplementary Figure 3 (b), while the transverse velocity in Supplementary Figure 3 (f) now changes direction with the change in rotation direction. Together these results clearly show that transverse velocity direction is directly related to the direction of rotation.

When the static magnetic field was increased in 0.2 mT increments, the exact static field at which the propulsion direction flips is revealed to be variable between different microparticles. While the averaged velocities were shown in Fig. 3 (c) of the main text, the individual trials can be seen in Supplementary Figure 4 (a), with each microparticle flipping direction at different static magnetic fields. We attribute variations between each trial to heterogeneities present within the fluid and conclude that the switching field also depends on the microparticle's environment. To complement the 0.2 mT incrementation shown in Fig. 3 (e) of the main text, the effect of a 1 mT increment on the same microparticle is shown in Supplementary Figure 4 (b) over 7 trials. For 1 mT increments, the switching fields of the hysteresis are at -1 mT and 1 mT, while for the 0.2 mT increments shown in the main text [Fig. 3 (e)] they are -1.2 mT and 0.8 mT, respectively.

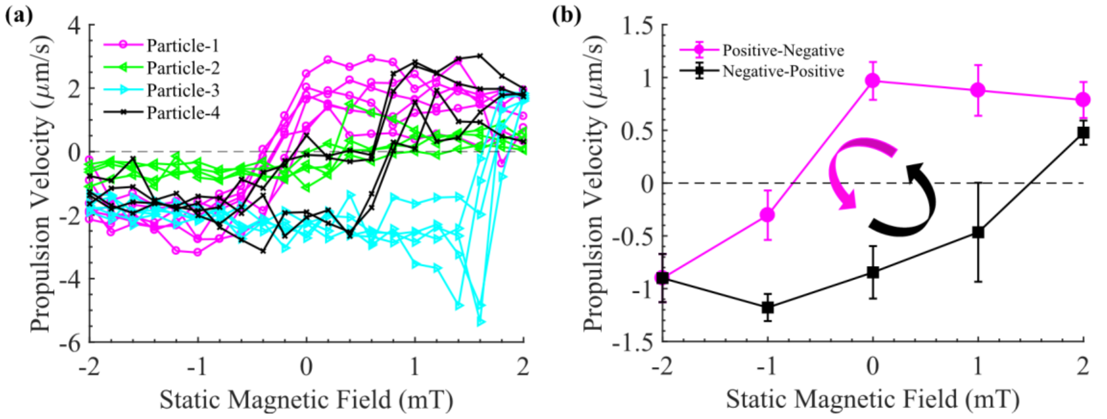

Supplementary Fig. 4. Comparing velocity profiles under different trials and field increments. (a) Individual trials for microparticles investigated in Fig. 3 (c) of the manuscript. The static magnetic field flux density necessary to cause a flipping of the propulsion direction was dependent on the specific microparticle as well as its local environment. (b) The hysteresis under 1 mT incrementation for the same microparticle as in Fig. 3 (e). Rotational frequency was 15 Hz in both cases. Data are presented as mean values +/- standard error. Seven independent trials were performed for the microparticle in (b). Source data are provided as a source data file.

It was observed during experiments that the frequency at which the microparticles rotated affected when their propulsion direction would flip during static field incrementation. In all experiments performed in mucin solution presented in the main text, the microparticles were rotated at a constant frequency of 15 Hz as the static magnetic field was incremented. Here, we describe comparative experiments where the frequency was reduced to 10 Hz. The propulsion velocity exhibited by the microparticle was lower and the behavior of the hysteresis curve from the static field sweep was significantly altered. Supplementary Figure 5 shows the static magnetic field incrementation from 2 mT to -2 mT to 2 mT under both 0.2 mT and 1 mT increments for two different rotational frequencies for the same microparticle [a different microparticle than main text Fig. 3 (e) and Supplementary Figure 4 (b)], over at least 4 trials per frequency and increment. Thus, rotation frequency can influence the microparticle's response to changing static magnetic fields. We expect that there are heterogeneities within the fluid medium that are also influencing the microparticles behavior, but these are not easily quantifiable at this time.

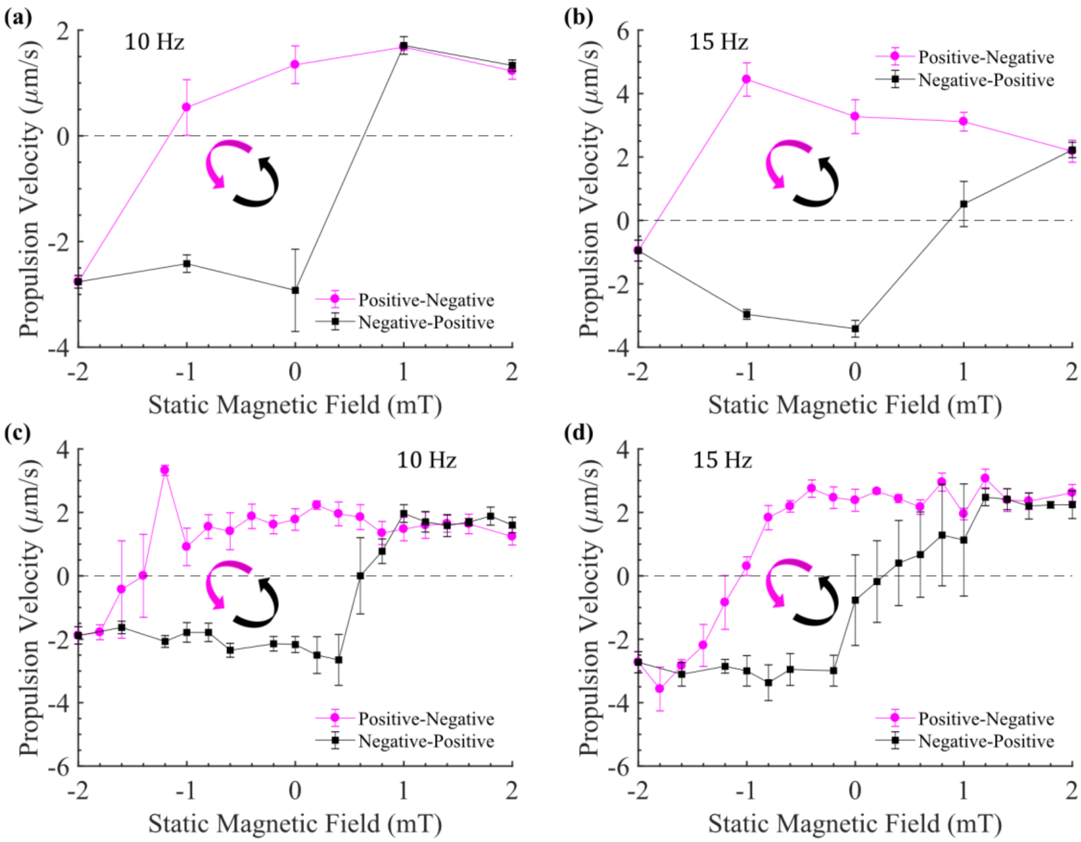

Supplementary Fig. 5. Hysteresis of a microparticle at different frequencies. The propulsion velocity vs. static magnetic field for a 10 µm diameter microparticle, under 1 mT increments from 2 to -2 to 2 mT, while rotating at (a) 10 Hz and (b) 15 Hz. The propulsion velocity vs. static magnetic field for the same 10 µm diameter microparticle under 0.2 mT increments, while rotating at (c) 10 Hz and (d) 15 Hz. The crossover points (where propulsion of the microparticle flips direction) in each graph were (a) -1 and 1 mT, (b) -2 and 1 mT, (c) -1.2 mT and 0.6 mT, and (d) -1.2 and approximately 0 mT. Data are presented as mean values +/- standard error. Four trials were performed for both microparticles at both frequencies and increments. Source data are provided as a source data file.

Static field experiments involving microparticles within 0.25% polyacrylamide did not always result in a hysteresis. This appears to be a frequency dependence as microparticles actuated at 15 Hz could not produce a hysteretic effect, while microparticles actuated at 40 Hz [see main text Fig. 3 (f)] could produce uniform and repeatable behavior. For five microparticles rotated at 15 Hz (4 trials each), no hysteresis was observed, and the behavior was similar to the unidirectional static sweeps in main text Fig. 3 (d). At higher frequencies (40 Hz and $|B_r| = 0.1750f$, where f is the frequency in Hz) hysteresis effects were observed in 4 of 5 particles tested, and for these particles two symmetry breaking propulsion states occur at 0 mT. Supplementary Figure 6 shows four microparticles, one under a 2 mT increment, one under a 1 mT increment, and two under 0.2 mT increments, each with at least three trials each. In Supplementary Figure 6 (a-b) we see there are two distinct propulsive states at 0 mT for each particle that are nearly equal and opposite to each other during hysteresis. In Supplementary Figure 6 (c-d) we see that the hysteresis patterns are different for different particles under 0.2 mT increments and were consistent between multiple trials; both also had two unique propulsive states for 0 mT. Thus, consistent with our observations in mucus solution, frequency, and static field incrementation are interlinked and can influence how

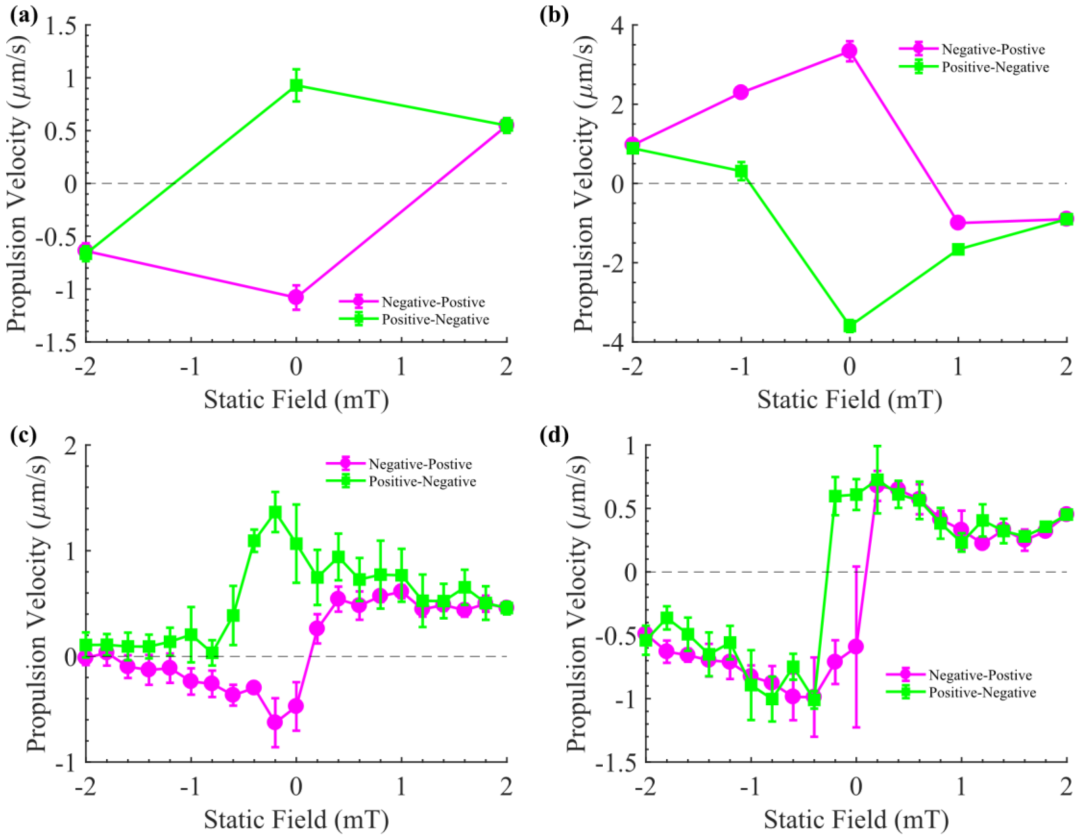

Supplementary Fig. 6. Hysteresis in 0.25% polyacrylamide. (a-b) Microparticles undergoing a static field hysteresis at 2 mT and 1 mT increments, respectively. (c-d) Two different microparticles undergoing a static field hysteresis at 0.2 mT increments. All microparticles were 10 μm in diameter and rotated at 40 Hz. Data are presented as mean values +/- standard error. The microparticle examined in (a) had seven independent trials, while the microparticles in (b-d) had three independent trials. Source data are provided as a source data file.

the hysteresis forms (if at all). However, even at a high frequency, some particles did not exhibit two symmetry breaking states; an example of this can be seen in Supplementary Figure 7, where a microparticle in 0.25% polyacrylamide had the same propulsion direction at 0 mT static magnetic field on both the forward and backward sweeps.

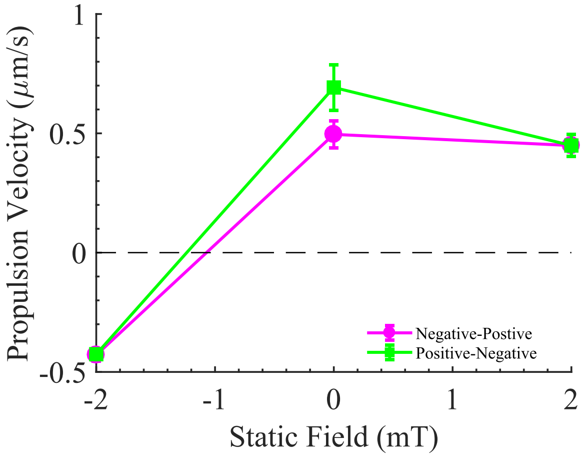

Supplementary Fig. 7. Attempted hysteresis of a 10 μm diameter microparticle in 0.25% polyacrylamide. This microparticle did not display hysteresis or pair of symmetry broken states when rotated at 40 Hz while magnetic field was changed in 2 mT increments. Data are presented as mean values +/- standard error. The microparticle examined here had three independent trials. Source data are provided as a source data file.

Supplementary Note 3: Performance Statistics of Selected Trajectories

Microparticles were propelled in the trajectories presented in the main text [Fig. 2 (c-g)] using proportional feedback control described in Eq. 4 and Eq. 5 of the main text. In the case of these experiments, we targeted two box-shaped trajectories for the microparticles in polyacrylamide, and the letters 'S','M'', and 'U' were targeted, in homage to Southern Methodist University, for microparticles in 4% mucin. Each of the trajectories was defined by several target positions which the microparticle had to reach in order to complete the trajectory. Connecting all of these target positions together in sequence creates the shortest path the microparticles could trace; these paths can be seen as magenta dashed lines in Fig. 2 (c-g) with the magenta-colored dots representing the target positions. The distance between the microparticle's centroid and the target points was measured during each frame of the recorded experiment to produce the error statistics presented in Supplementary Figure 8 (a-e) for Fig. 2 (c-g), respectively. In all experiments the error from the target points decayed and the microparticles could come reasonably close to each target position (less than 5 μm from target).

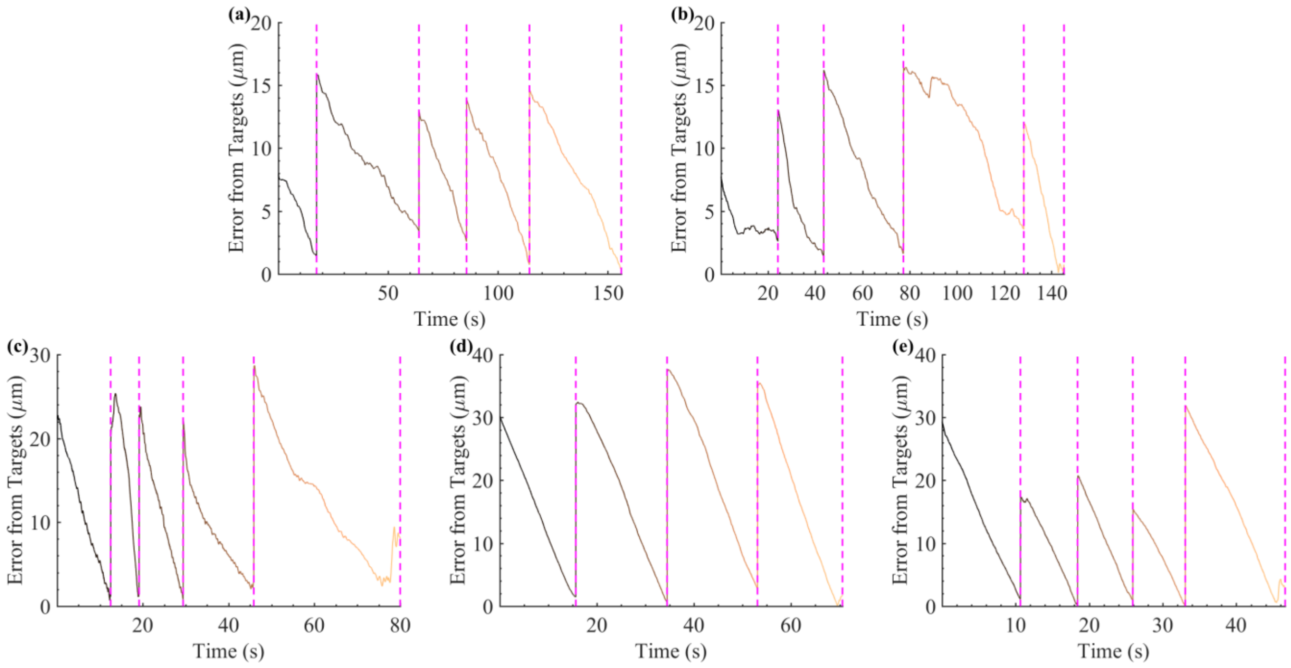

Supplementary Fig. 8. Feedback control trajectories and performance statistics. (a-e) Represent the error statistics for trajectories shown in Fig. 2 (c-g) of the main text, respectively. The dashed magenta lines in (a-e) indicate the target point the microparticle was attempting to reach.

Supplementary Note 4: 3D Propulsion and Tracking

To approximate the displacement of a microparticle translating in 3D, two different methods were utilized: (1) directly measuring using a microscope focusing knob and (2) measuring the change in pixel area of a microparticle as it changes focal planes. The first method was used to measure a microparticle's distance and velocity as it translated from the bottom of the substrate into the bulk fluid during the µPIV experiments discussed in the manuscript. The microscope was a Nikon Eclipse-Ti which could precisely track the focal plane in micrometers via a visual display at the front of the microscope. A microparticle, the same one that was examined in the µPIV experiment in main text Fig. 4 (i), was rotated for 10 seconds in the *x-y* plane at 15 Hz with a 2 mT static magnetic field using a MagnetbotiX (MFG-100-i) field controller; the distance traveled by the microparticle was recorded from which the velocity was calculated. The results of this experiment are shown in Supplementary Figure 9 (b), where the bar graphs show both the distance traveled and the average velocity of the microparticle. The microparticle was clearly seen to overcome gravity and achieve meaningful propulsion behavior in a short time frame. In more complex pathing experiments, a Leica (DM IRB) microscope was utilized with our approximate Helmholtz coil system and a CMOS camera was used to visualize the microparticles as they translated through the medium. These microparticles were tracked in the *z*-direction by tracking changes to the pixel area of the observed microswimmer; decreases in pixel area were correlated with decreases in *z*-direction depth (into the page), and increases were correlated with increase in *z*-direction depth (out of the page). A graph showing this correlation can be seen below in Supplementary Figure 9 (a). This correlation was used to generate the positional data for the results shown in Fig. 2 (h) of the main text from pixel areas in the videos. Another correlation for a 2 µm diameter microparticle in 0.25% polyacrylamide can be seen in Supplementary Figure 9 (c).

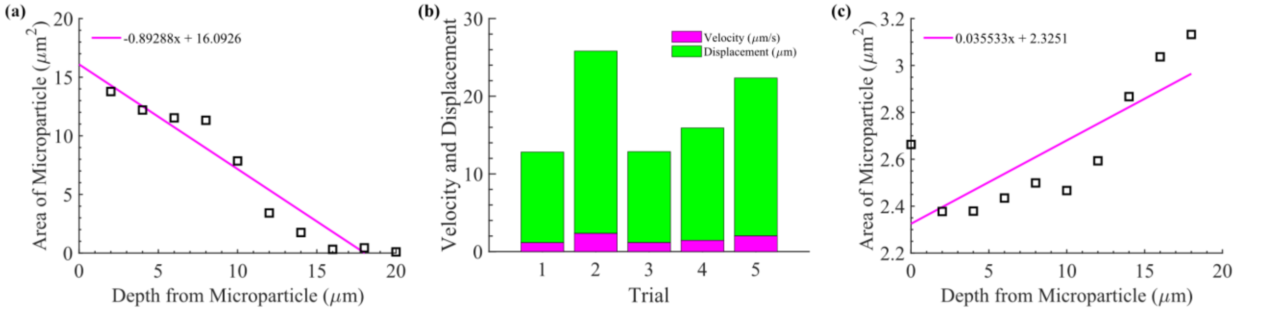

Supplementary Fig. 9. 3D tracking analysis. (a) Image area of a microparticle as the microparticle translates in the negative *z*-direction. (b) Velocity and distance measured for a microparticle propelling along the positive *z*-direction. (c) Image area as a 2 µm diameter microparticle translates in the negative *z*-direction in 0.25% polyacrylamide.

While not shown in the main text, microparticles suspended in 0.25% polyacrylamide were also capable of exhibiting 3D propulsive behavior similar to the kind demonstrated in 4% mucin [main text, Fig. 2 (h)]. From experiments it was determined that 2 μm diameter microparticles were not as susceptible to gravity driven sedimentation and were used here for demonstrative purposes. Presented in Supplementary Figure 10 is a 2 μm diameter microparticle that performed a user selected trajectory in three dimensions with *z*-position approximated using the correlation shown in Supplementary Figure 9 (c). The estimated 3D position with time is also presented along with projections in each plane.

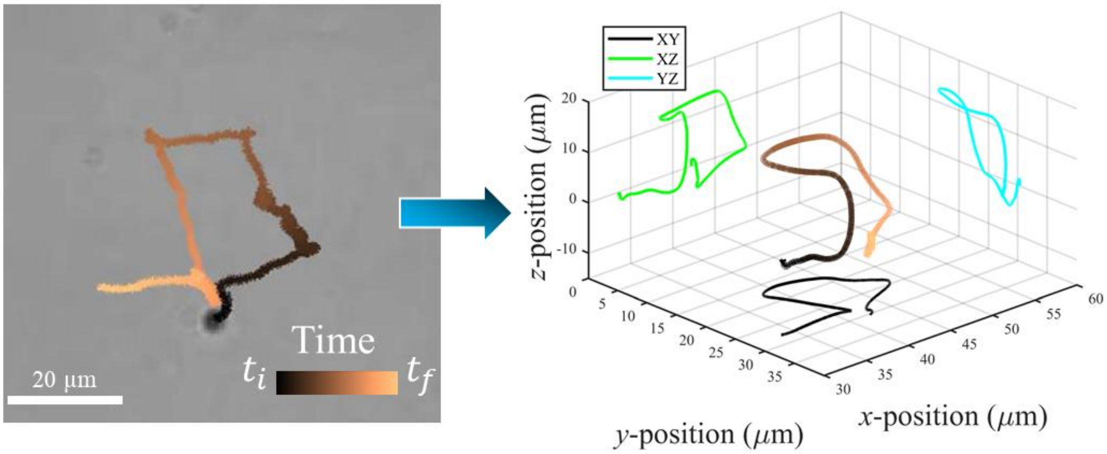

Supplementary Fig. 10. Three-dimensional tracking of a 2 μm diameter bead in 0.25% polyacrylamide. Total time of experiment was 138 seconds. This was repeatable for greater than 90% of all microparticles examined at this diameter.

Supplementary Note 5: Rotational Dynamics of Magnetic Spheres with Static Field

Consider a magnetic field that is rotating about the *x*-axis, with static component B_s and rotating component B_r. We work using a basis that is coincident and co-rotating with the magnetic field, so that the magnetic field is $\mathbf{B} = (B_s, B_r, 0)$, and its angular velocity is $\boldsymbol{\omega} = (\omega,0,0)$. For a spherical bead with magnetic moment of magnitude m, $\mathbf{m} = m(\hat{m}_x, \hat{m}_y, \hat{m}_z)$, the torque exerted on it by the field is $\mathbf{N} = \mu_0 \mathbf{m} \times \mathbf{B}$, where μ_0 is the vacuum magnetic permeability. In the zero-Reynolds number limit, the angular velocity $\boldsymbol{\Omega}$ of a sphere of radius a is proportional to the torque, $\boldsymbol{\Omega} = \frac{3}{8\pi\mu a^3}\mathbf{N}$, where μ is the viscosity of the fluid. Combining the above, $\boldsymbol{\Omega} = \frac{3\mu_0}{8\pi\mu a^3}\mathbf{m} \times \mathbf{B}$.

Suppose we seek a steady solution, in which the sphere rotates with the field, i.e., $\boldsymbol{\Omega} = \boldsymbol{\omega}$. Since by the properties of the cross product $\boldsymbol{\Omega}$ is perpendicular to $\mathbf{B}$, this is only possible if $\boldsymbol{\omega}$ is perpendicular to $\mathbf{B}$, i.e., if there is no static component and $B_s = 0$. However, we are interested in the behavior when there is a static component, so we must seek a different solution. It turns out that for $B_s \neq 0$, there exist solutions for which the magnetic dipole $\mathbf{m}$ co-rotates with the field, even if the sphere does not. To find these, we set the time evolution of $\mathbf{m}$ in the basis rotating with the field to be zero, $0 = \dot{\mathbf{m}} = (\boldsymbol{\Omega} - \boldsymbol{\omega}) \times \mathbf{m}$, where the relative angular velocity $(\boldsymbol{\Omega} - \boldsymbol{\omega})$ is used since we are expressing $\mathbf{m}$ in the basis rotating with angular velocity $\boldsymbol{\omega}$. The result is

$$\dot{\mathbf{m}} = m\Omega_s \begin{pmatrix} b_s(1-\widehat{m}_x^2) - \widehat{m}_x\widehat{m}_y \\ -b_s\widehat{m}_x\widehat{m}_y + (1-\widehat{m}_y^2) \\ -b_s\widehat{m}_x\widehat{m}_z - \widehat{m}_y\widehat{m}_z \end{pmatrix} + m\omega \begin{pmatrix} 0 \\ \widehat{m}_z \\ -\widehat{m}_y \end{pmatrix}, \qquad (1)$$

where $b_s = B_s/B_r$, and $\Omega_s = \frac{3mB_r\mu_0}{8\pi a^3\mu}$ is the step-out frequency of the sphere when there is no static field.

Solutions for a co-rotating dipole **m** are obtained by setting Supplementary Equation (1) to zero and solving under the constraint $1 = \widehat{m}_x^2 + \widehat{m}_y^2 + \widehat{m}_z^2$. Typically, there are four solutions, only two of which have purely real components $(\widehat{m}_x, \widehat{m}_y, \widehat{m}_z)$. One of these real solutions has $\widehat{m}_x$ pointing in the direction of the static component B_s, and the other has $\widehat{m}_x$ pointing in the opposite direction of B_s.

To determine which of these solutions is observed, we evaluate their stability. Denote the direction of the dipole for the solution in question $\widehat{\mathbf{m}}_0$, which satisfies $\dot{\mathbf{m}}(\widehat{\mathbf{m}}_0) = 0$. Since $\widehat{\mathbf{m}}_0$ is a unit vector, it has only two degrees of freedom, which we can express in terms of an infinitesimal rotation $\boldsymbol{\sigma}$, such that a perturbation $\delta\widehat{\mathbf{m}}_0 = \boldsymbol{\sigma} \times \widehat{\mathbf{m}}_0$, where it is sufficient to consider $\boldsymbol{\sigma}$ in the two-dimensional space perpendicular to $\widehat{\mathbf{m}}_0$. Then we find that $\dot{\boldsymbol{\sigma}} \times \widehat{\mathbf{m}}_0 = \dot{\mathbf{m}}(\widehat{\mathbf{m}}_0 + \delta\widehat{\mathbf{m}}_0)$, or expanding to first order in the perturbation, $\dot{\boldsymbol{\sigma}} \times \widehat{\mathbf{m}}_0 = -\frac{\partial\dot{\mathbf{m}}}{\partial\widehat{\mathbf{m}}}(\widehat{\mathbf{m}}_0 \times \boldsymbol{\sigma})$. From this we obtain

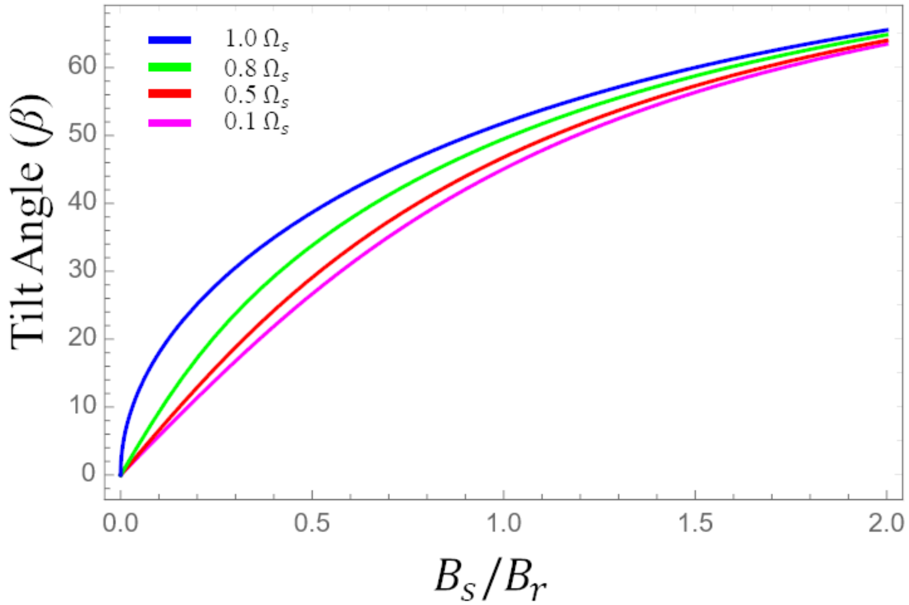

Supplementary Fig. 11. Change in tilt angle in response to applied static magnetic field component B_s. Each curve is produced for the indicated value of rotation frequency (ω).

$$\dot{\boldsymbol{\sigma}} = -\hat{\mathbf{m}}_0 \times \left(\frac{\partial \dot{\mathbf{m}}}{\partial \hat{\mathbf{m}}}(\hat{\mathbf{m}}_0 \times \boldsymbol{\sigma})\right) \equiv \mathbf{Q}\boldsymbol{\sigma}, \quad (2)$$

where the last equivalence serves to define the linear operator **Q**. To be explicit, in indicial notation

$$Q_{ij} = -\epsilon_{ikl}\hat{m}_k \frac{\partial \dot{m}_l}{\partial \hat{m}_n} \epsilon_{npj}\hat{m}_p, \quad (3)$$

where all the components are evaluated at $\hat{\mathbf{m}}_0$, and ϵ_{ijk} is the Levi-Civita antisymmetric tensor. **Q** has one zero eigenvalue (corresponding to the $\hat{\mathbf{m}}_0$ direction) and two other eigenvalues. The solution $\hat{\mathbf{m}}_0$ is stable if the two other eigenvalues both have negative real parts. We find that the solution with $\hat{m}_x$ pointing in the direction of the static component B_s is stable, and the other is unstable.

Physically, the kinematics of the stable solution involves the magnetic dipole tilting towards the static component of the magnetic field, the sphere rotating about the *x*-axis such that the magnetic dipole rotates at the same rate as the rotating field, and the sphere additionally rotating about the magnetic dipole direction (which is itself changing in the lab frame). Thus, only the magnetic dipole undergoes steady rotation at the field angular velocity, while the sphere itself undergoes a more complex time-dependent rotation. In Supplementary Figure 11, we plot the tilt angle β between the *y*-*z* plane and the dipole ($\hat{m}_x = \sin\beta$) for increasing static field, and various field rotation rates. For zero static field, $\beta = 0$, but as the static field component increases so does the tilt angle.

Supplementary Note 6: Symmetry Breaking Propulsion Force

The force on a simultaneously rotating and translating sphere in the zero Reynolds number limit in a nonlinear third-order fluid was calculated using a retarded motion expansion[1] by Giesekus[2]. For a sphere translating in the *z*-direction and rotating with angular velocity Ω about the *z*-axis, up to second order in Deborah number the force in the *z* direction takes the form:

$$F = -6\pi\mu a U + D U^3 + C\Omega^2 U, \quad (4)$$

where *D* and *C* are constants that depend on the material parameters of the fluid. As discussed in the main text, the term proportional to $U\Omega^2$ couples rotation to translation in a way that corresponds to a possible symmetry-breaking force. If $C > 0$, the nonlinear force opposes drag and is propulsive, while if $C < 0$ the nonlinear force increases drag and stabilizes the state with no translation. Thus, we are interested in whether *C* is positive or negative for various nonlinear fluids. The results for various nonlinear fluids are discussed below.

Giesekus[2] calculates the force for both a dilute solution of elastic dumbbells, i.e., an Olroyd-B fluid, (his Eq. 72) and a dilute solution of rigid dumbbells or rods (his Eq. 74), and finds positive *C* for both. (Note that the sign of Giesekus' force in his paper is opposite ours since his sphere translates and rotates in the opposite directions.)

Table 6.2-1 of the textbook of Bird, Armstrong and Hassager[1] contains a table listing the parameters for the constitutive laws of third-order fluids corresponding to various kinetic theory models, namely a dilute solution of FENE dumbbells, a dilute solution of multi-bead rods, and a melt of freely-joined bead-rod chains. The table reports the coefficients b_1, b_2, b_{11}, b_3, b_{12}, and $b_{1:11}$ of the constitutive law, Eq. 6.2-1 of that textbook, which is written in terms of the rate of strain tensors defined therein (denoted $\gamma_{(n)}$in Bird, Armstrong and Hassager[1]). In order to use these parameters in Giesekus' result, one must deduce from them the values of the coefficients denoted $\gamma_0^{(1)}$, $\gamma_0^{(2)}$, $\gamma_0^{(11)}$, $\gamma_0^{(3)}$, $\gamma_0^{(21)}$, and $\gamma_{11}^{(1)}$ in the constitutive law Eq. 3 (from Giesekus[2]) which is written in terms of (different) kinematic tensors $f^{(n)}$ defined in that paper. We found that

$$2f^{(1)} = \gamma_{(1)},$$
$$2f^{(2)} - 4f^{(1)}\bullet f^{(1)} = \gamma_{(2)}, \tag{5}$$
$$2f^{(3)} - 6\left(f^{(1)}\bullet f^{(2)} + f^{(2)}\bullet f^{(1)}\right) + 8(f^{(1)}\bullet f^{(1)}\bullet f^{(1)}) = \gamma_{(3)},$$

and that if

$$\begin{pmatrix} \gamma_0^{(1)} \\ \gamma_0^{(2)} \\ \gamma_0^{(11)} \\ \gamma_0^{(3)} \\ \gamma_0^{(21)} \\ \gamma_{11}^{(1)} \end{pmatrix} = \begin{pmatrix} 2 & 0 & 0 & 0 & 0 & 0 \\ 0 & 2 & 0 & 0 & 0 & 0 \\ 0 & -4 & 4 & 0 & 0 & 0 \\ 0 & 0 & 0 & 2 & 0 & 0 \\ 0 & 0 & 0 & -6 & 4 & 0 \\ 0 & 0 & 0 & 4 & -8 & 8 \end{pmatrix} \begin{pmatrix} b_1 \\ b_2 \\ b_{11} \\ b_3 \\ b_{12} \\ b_{1:11} \end{pmatrix}, \tag{6}$$

then the constitutive laws given by Bird, Armstrong and Hassager[1] and Giesekus[2] were identical up to an isotropic function that could be absorbed into the pressure. Using this conversion, we find the nonlinear corrections of the force due to the constitutive laws in the table.

For the melt of freely-jointed bead-rod chains, we tested the sign of *C* at room temperature for molar volumes of polymer molecules of approximately[3] 10^{-4} m^3/mol, corresponding to number densities of approximately 10^{28} molecules/m^3, a range of number of beads in each chain of 2 – 10, a solvent viscosity of 1 mPa, a range of time constants λ^k in Table 6.2-1 of Bird, Armstrong and Hassager[1] of 0.1-100 s, and a range of ε of 0.3-0.5 as suggested in that table. In all cases *C* was positive.

For the dilute solution of FENE dumbbells, we tested the sign of *C* at room temperature for number densities of polymer molecules in the range of 10^{26} – 10^{28} molecules/m^3, a solvent viscosity of 1 mPa, a range of time constants λ_H in Table 6.2-1 of Bird, Armstrong, and Hassager[1] 0.1-100 s, and a range of *b* of 10-300 as suggested in that table. In all cases *C* was positive.

For the dilute solution of multibead rods, we tested the sign of *C* at room temperature for number densities of polymer molecules in the range of 10^{26} – 10^{28} molecules/m^3, a solvent viscosity of 1

mPa, a range of time constants $\lambda_N^{(1)}$ of 0.1-100 s, and a range of $[1-\lambda_N^{(2)}/\lambda_N^{(1)}]$ of -0.5 – 0.3082 in Table 6.2-1 of Bird, Armstrong, and Hassager[1] as suggested in the that table. In all cases *C* was positive.

To summarize, in all the cases tested, the sign of *C* was positive, which indicates that the nonlinear correction to the force around a translating and rotating sphere acts to propel it, at least up to the order of the retarded motion expansion analyzed. As discussed in the main text, this perturbative analysis is only suggestive and cannot explicitly find the symmetry-broken state. An explicit theoretical solution demonstrating symmetry breaking would likely require numerical simulations of a sphere in a nonlinear fluid.

Supplementary Note 7: Approximate Helmholtz Coils and Magnetic Field Spatial Dependence

All experiments except those involving μPIV were conducted using an approximate Helmholtz coil system. This system has been used extensively in our previous research[4-6]. The approximate Helmholtz coils themselves are composed of AWG 25 copper wire with approximately 600 turns each, have an outer diameter of 6.55 cm, an inner diameter of 4 cm, and a thickness of approximately 1.23 cm. The coils are separated from each other at a distance of twice their radius, differing from a traditional Helmholtz coil which has 1 radius of separation (thus why we call them 'approximate'). To account for this discrepancy, the voltages provided to the coils are increased such that a uniform magnetic field profile is present in the center of each coil pair. The coils are arranged in a tri-axial arrangement allowing for approximately uniform magnetic fields to be produced in all three dimensions. More details about specific experimental procedures can be found in the main text.

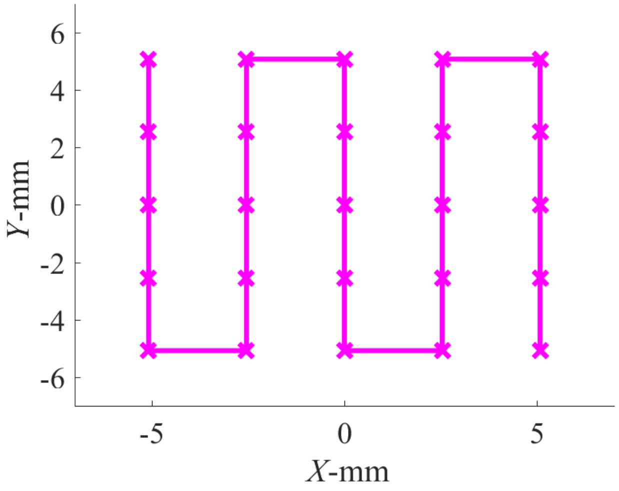

Supplementary Fig. 12. Path of the magnetometer within the working space of the approximate Helmholtz coil system. The probe of the magnetometer began at (-5, 5) and ended at (5, -5) with each point marked by an '×' representing a measurement location.

A THM1176 three axis Hall magnetometer (GWM Associates) was used to measure the magnetic fields at predefined locations within the approximate Helmholtz coil system. The working space at the center of the coil system was confined to an area of 2 cm^2; within this area the teslameter was moved at 2 mm increments along the path designated in Supplementary Figure 12. Measurements along this path were taken at *Z*-heights of 0, 1, and 2 mm for each coil pair. During experiments only one pair of coils was set to produce a static field of 2 mT. The measured magnetic fields at each point and height are displayed as a gradient heat map in Supplementary Figure 13 for all three coil pairs. In each plane the magnetic fields are mostly uniform, although they are slightly higher than the expected 2 mT value. The *Z* coil pair [Supplementary Figure 13 (c)] appears to have high variation in magnetic fields as height increases along the *Z*-axis; this is confirmed when comparing the static field distributions of all three planes for each coil pair [Supplementary Figure 13 (d-f)]. However, the maximum magnetic gradient does not exceed 0.05 mT/mm. Furthermore, given the evidence in the main text regarding the hysteresis of both microparticles suspended in mucus and polyacrylamide having noticeable propulsive behavior at 0 mT under purely rotating magnetic fields, we can again dismiss gradients along the propulsive direction as being the primary driver of microparticle propulsion. The control data sets in Fig. 1 (b) involving 15% NaCl and 0.2% methylcellulose also demonstrate that propulsion is not occurring even at high rotational frequencies.

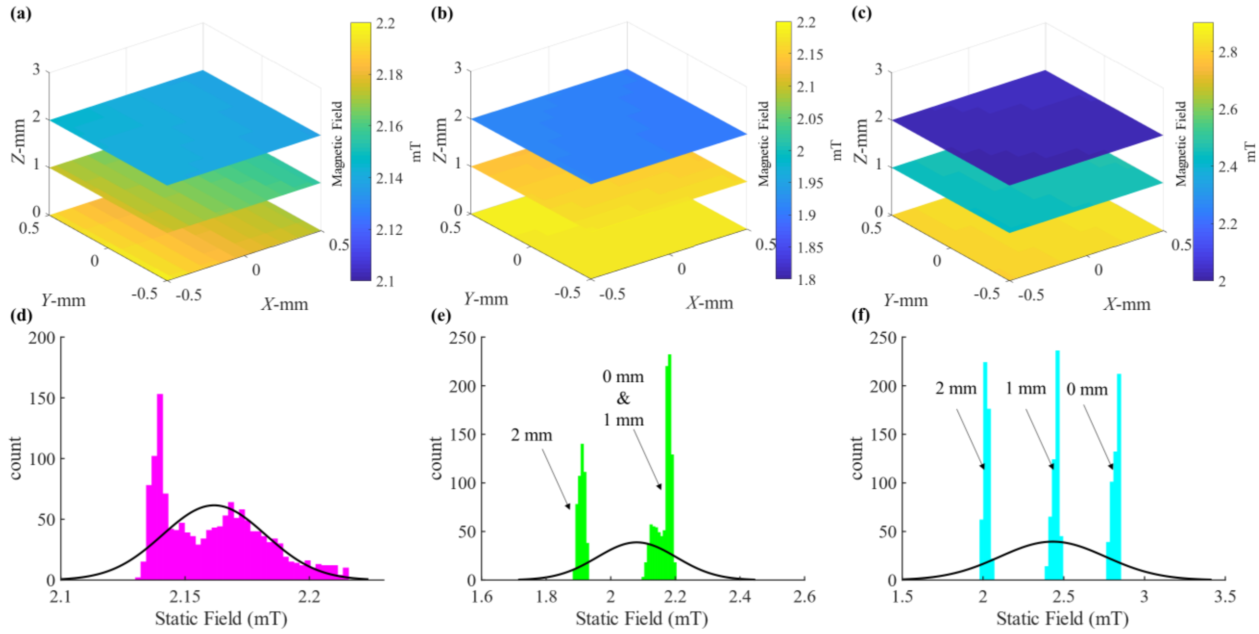

Supplementary Fig. 13. Approximate Helmholtz coil magnetic field profiles. Magnetic field profiles for (a) *X* coil, (b) *Y* coil, and (c) *Z* coil at different spacings in the *XYZ* plane under a constant 2 mT static field along each respective direction for (a-c). (d-f) Static field value histograms of all three planes of measurement with estimated normal distributions (black solid lines). The mean static fields were (d) 2.16 mT, (e) 2.08 mT, and (f) 2.43 mT, while the standard deviations were 0.02 mT, 0.12 mT, and 0.34 mT, respectively. The *Z* coils had significant variance in static field values as height varied, while *Y* coil had a slight variance.

Supplementary Note 8: Magnetic Field Gradients are not Responsible for Propulsion

In the main text we remark that since propulsion disappears as the rotational frequency approaches zero even though the static field remains present, gradients in the static magnetic field cannot be responsible for the observed propulsion. In this section we provide additional evidence ruling out magnetic field gradients as the cause of propulsion.

First, the estimated force on a magnetic bead was estimated to be 8×10^{-14} N, using the estimated two dimensional dipole moment of $[-1.899 \times 10^{-12}, \quad 6.277 \times 10^{-13}]$ A.m^2 produced from the maximum magnetic field values of $[1.6978, \quad 1.6733]$ mT measured in the *x*-*y* plane; using Stokes' law this force results in an estimated translational velocity of 0.0046 μm/s for a 10 μm diameter bead.

Since the estimated translational velocity is order of magnitudes smaller than typically observed propulsion velocities (of order 1 μm/s), the gradient field cannot be responsible for the propulsion observed. In addition, the static magnetic field gradients could not account for the demonstrated controllable propulsion along different directions.

To further assure that magnetic field gradients were not causing the translation behavior, microparticles of 10 μm diameter were dispersed inside a sample chamber containing a 4% mucin synthetic mucus medium and placed inside the approximate Helmholtz coil system. A 2 mT static field was applied along the X-coil pair (positive or negative) and microparticles were examined under both rotating (15 Hz) and non-rotating conditions for at least 20 seconds and videos were recorded at 30 frames per second (fps). A mean square displacement (MSD) analysis was performed to compare the diffusive behavior of microparticles under both conditions. The MSD was calculated using,

$$< r^2(\tau) > = \frac{1}{N-\tau} \sum_{i=1}^{N-\tau} [r(t_i + \tau) - r(t_i)]^2, \quad (7)$$

where $< r^2(\tau) >$ is the MSD, N is the number of time steps for a given trajectory, τ is the lag time, r is the position vector, and t_i is the ith time increment[7]. For both the rotating and non-rotating cases the MSD profiles were modeled using,

$$< r^2(\tau) > \propto 4D\tau^{\alpha}, \quad (8)$$

where D is generalized diffusion and α is the anomalous diffusion exponent; fittings were produced from the ensemble averaged MSD data over multiple rotating microparticles (at least three with each having at least three independent trials) and non-rotating microparticles under the same conditions. The MSD of the rotating and non-rotating microparticles (averaged over at least 3 beads, each with at least 5 trials) can be seen in Supplementary Figure 14 (a-b). On the non-logarithmic plot [Supplementary Figure 14 (a)] the rotating behavior clearly induces ballistic behavior while the non-rotating case retains a flat MSD throughout. Comparing the results in Supplementary Figure 14 (b) against diffusive (α~1) and superdiffusive behavior (α > 1), it is clear that rotating microparticles are actively propelling under rotation, while the non-rotating microparticles are only experiencing approximately diffusive behavior. After fitting the MSD data between lag time ranges of 1 to 5 seconds using a Delayed Rejection Adaptive Metropolis Markov

chain Monte Carlo technique[8], (see SI Supplementary Note 11 for more information) we find that diffusivity and the diffusion exponent are both reasonable for each case. For the non-rotating case the diffusivity and diffusion exponent were 0.0019 ($\frac{\mu m^2}{s^\alpha}$) and 1.05. The rotating case had a diffusivity and diffusion exponent of 0.44 ($\frac{\mu m^2}{s^\alpha}$) and 1.74; the plots for each can be seen as solid lines in Supplementary Figure 14 (b).

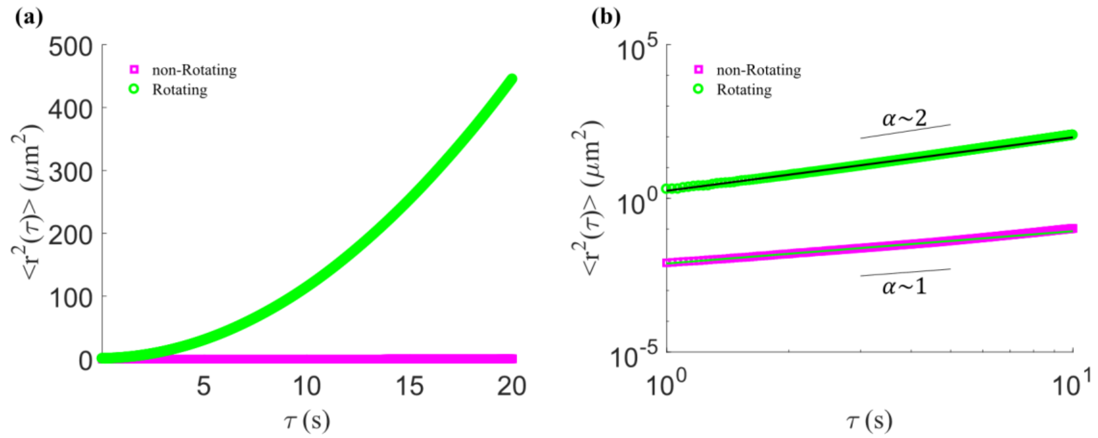

Supplementary Fig. 14. Mean square displacement of rotating and non-rotating microparticles in 4% mucin. Mean square displacement where (a) is the non-logarithmic plot and (b) shows the logarithmic plot with fittings. The black long solid line corresponds to the fit for the rotating case and the long solid green line is the fit for the non-rotating case. Short solid lines represent general mean square displacement profiles for different values of α. Source data are provided as a source data file.

Supplementary Note 9: Propulsion Velocity Dependence on Mucin Concentration and Particle Size

Only synthetic mucus of 4% mucin was explored in detail within the main text and can be considered a reasonable baseline since mucin concentrations within the human body typically ranges between 2-5%[9]. However, it was observed experimentally that microparticles could exhibit similar propulsive behavior for other concentrations of mucins. Presented in Supplementary Figure 15 are the propulsion velocities of 10 µm diameter microparticles within 2%, 3%, and 4% mucin solutions at different rotational frequencies; all data sets were averaged over at least six microparticles per diameter, per fluid, with at least 3 trials each. While at small frequencies (less than 10 Hz) there is only minor variation between propulsion velocities, at larger frequencies (greater than 10 Hz) there are clear differences in propulsion behavior. Based on these trends it appears that increasing mucin concentration positively affects propulsion velocity, while lowering mucin concentration results in a velocity decrease. The limits of this behavior are not well understood at this time although it is expected that at extreme (higher or lower) mucin concentrations the microparticle propulsion velocity should decrease.

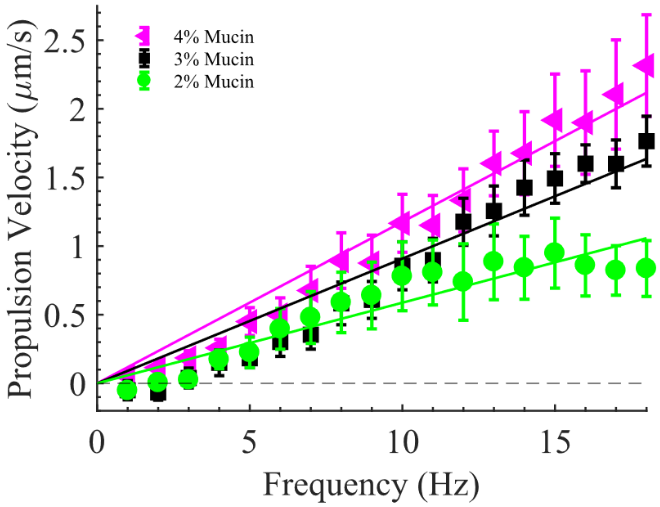

Supplementary Fig. 15. Velocity and mucin concentration. Propulsion velocity vs. frequency for 10 µm diameter microparticles inside different concentrations of mucin. Data are presented as +/- standard error. Statistics: six microparticles were examined in 2% and 4% mucin, while seven microparticles were examined in 3% mucin; there were at least 3 independent trials per microparticle. Source data are provided as a source data file.

We tested microparticles of several different diameters (2, 4, 8, and 10 µm) to determine if propulsion velocity was influenced by microparticle size [Supplementary Figure 16 (a-b)]; all data sets were averaged over at least three microparticles per fluid with at least 3 trials each. In 4% mucin as microparticle diameter increased, so did propulsion velocity, except that both 2 µm diameter microparticles and 4 µm diameter microparticles were nearly identical and had overlapping velocity curves. In 0.25% polyacrylamide the influence of microparticle diameter was less obvious, with 2 µm microparticles being faster than 4 µm microparticles, and 8 µm microparticles having a markedly non-linear relationship to frequency. Other than the 8 µm microparticles in polyacrylamide, for frequencies less than 10 Hz, the velocities of the differently sized microparticles in both fluids were all within a standard error of each other; but for frequencies

above 10 Hz the velocity profiles of each diameter microparticle become more distinct from each other.

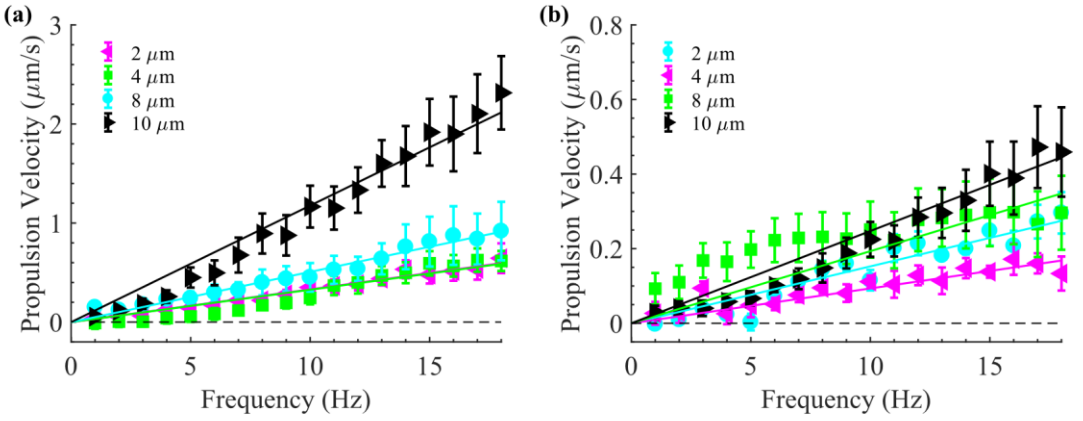

Supplementary Fig. 16. Velocity and microparticle diameter. (a) Propulsion velocity vs. frequency for different diameter microparticles in 4% mucin. The coefficients of determination (r^2) values were 0.976, 0.8875, 0.9733, and 0.9650 for 2 µm, 4 µm, 8 µm, and 10 µm, respectively. (b) Propulsion velocity vs. frequency for different diameter microparticles in 0.25% polyacrylamide. The r^2 values were 0.9255, 0.8066, -0.4007, and 0.9483 for 2 µm, 4 µm, 8 µm, and 10 µm, respectively. Data are presented as mean values +/- standard error. Statistics: (a) twelve, fifteen, three, and six microparticles were examined for 2, 4, 8, and 10 µm diameter microparticles, respectively; (b) six, six, eight, and seven microparticles were examined for 2, 4, 8, and 10 µm diameter microparticles, respectively. All microparticles had at least three independent trials. Source data are provided as a source data file.

Supplementary Note 10: Rheological Analysis of First and Second Normal Stress

The first normal stress difference (N_1) was measured directly from the axial force (F_{CP}) in a 2° cone-and-plate geometry (60 mm in diameter) on a TA Instruments AR-G2 rheometer,

$$N_1 = \frac{2F_{\text{CP}}}{\pi R^2}. \tag{9}$$

The second normal stress difference (N_2) was measured by comparing the axial force in the cone-and-plate geometry to the axial force (F_{PP}) in a parallel-plate (60 mm diameter) geometry[10,11],

$$N_1 - N_2 = \frac{F_{\text{PP}}}{\pi R^2}\left[2 + \frac{d\ln\left(\frac{F_{\text{PP}}}{\pi R^2}\right)}{d\ln(\dot{\gamma})}\right], \tag{10}$$

where R is the radius of the geometry, and $\dot{\gamma}$ is the shear rate at the rim of the geometry. We zeroed the axial force balance before each sweep of shear rates and checked that the meniscus of the sample did not change shape during the sweep to ensure that capillary forces did not affect the axial force measurements. A polymethyl methacrylate (PMMA) sheet was used for the lower geometry surface to stabilize the contact line. Care was taken to trim the gap after sample loading and to ensure no air bubbles were trapped in the rheometer during sample loading.

We validated our measurements by comparing measurements of the normal stresses of a 2.5% polyacrylamide solution in water [Supplementary Figure 17 (a), averaged over 3 runs] to those reported in the literature[12] (see Fig. 3.3-5 in reference). Similar to the reported values for 1.5% polyacrylamide solution in a water-glycerin mixture, we measured a positive first normal stress coefficient,

$$\Psi_1 = \frac{N_1}{\dot{\gamma}^2}, \quad (11)$$

that decreased with increasing shear rate as $\dot{\gamma}^{-\frac{3}{2}}$, and a negative second normal stress coefficient,

$$\Psi_2 = \frac{N_2}{\dot{\gamma}^2}, \quad (12)$$

that was about 23% the magnitude of the first normal stress coefficient [Supplementary Figure 17 (b)]. The magnitude of our measured normal stresses were systematically smaller than those reported previously, possibly due to differences in the solvents and molecular weight of the polyacrylamide.

We measured the normal stress differences of a 10% mucin solution for shear rates between 100 and 1000 s^{-1} (near microparticles rotating at 15-20 Hz, shear rates are 94.25-125.66 s^{-1} in our experiments). For each geometry, three runs were performed with the addition of a drop of nonionic surfactant (Nonidet P-40; 0.025 m/v% aqueous solution) to the air-sample interface. For these shear rates, inertial effects and normal stress differences contributed similar magnitude axial forces. We corrected for inertial effects in two different ways in Supplementary Figure 17 (c-e). The expected axial forces due to inertia are theoretically expected to be $F = -0.075\pi\rho W^2 R^4$ where ρ is the density of the fluid, W is the angular velocity, and R is the radius of the geometry[13]. Normal stress difference measurements corrected using these expected axial forces were used to plot Supplementary Figure 17 (c), while the normal stress coefficients were plotted in Fig. 5 (e). As a check of the accuracy of the theoretical inertial correction, we also measured axial forces due to inertia by performing measurements on water, with and without the addition of surfactant (Nonidet P-40; 0.025 m/v% aqueous solution) to account for any capillary effects. We observed no significant dependence on geometry type or impact due to capillary forces; however, we did find the measured contribution due to inertia to be slightly less than expected. Normal stress difference measurements and normal stress coefficients corrected using these measured axial forces were used to plot Supplementary Figure 17 (d-e).

Both main text Fig. 5 (e) and Supplementary Figure 17 (e) show that the mucin solution also has positive first normal stress coefficients and smaller negative second normal stress coefficients in this range of shear rates. The magnitude of normal stress differences is significantly smaller than those we measured in polyacrylamide solution. For both 0.25% polyacrylamide and 4% mucin solution, axial forces were even smaller and below the limit of the sensitivity of the force transducers on both rheometers used during experiments, so we could not obtain reliable measurements.

To summarize, our measurements show that mucin solutions develop normal stress differences under shear, which can lead to rod-climbing-like effects such as those we propose are responsible for symmetry-breaking propulsion.

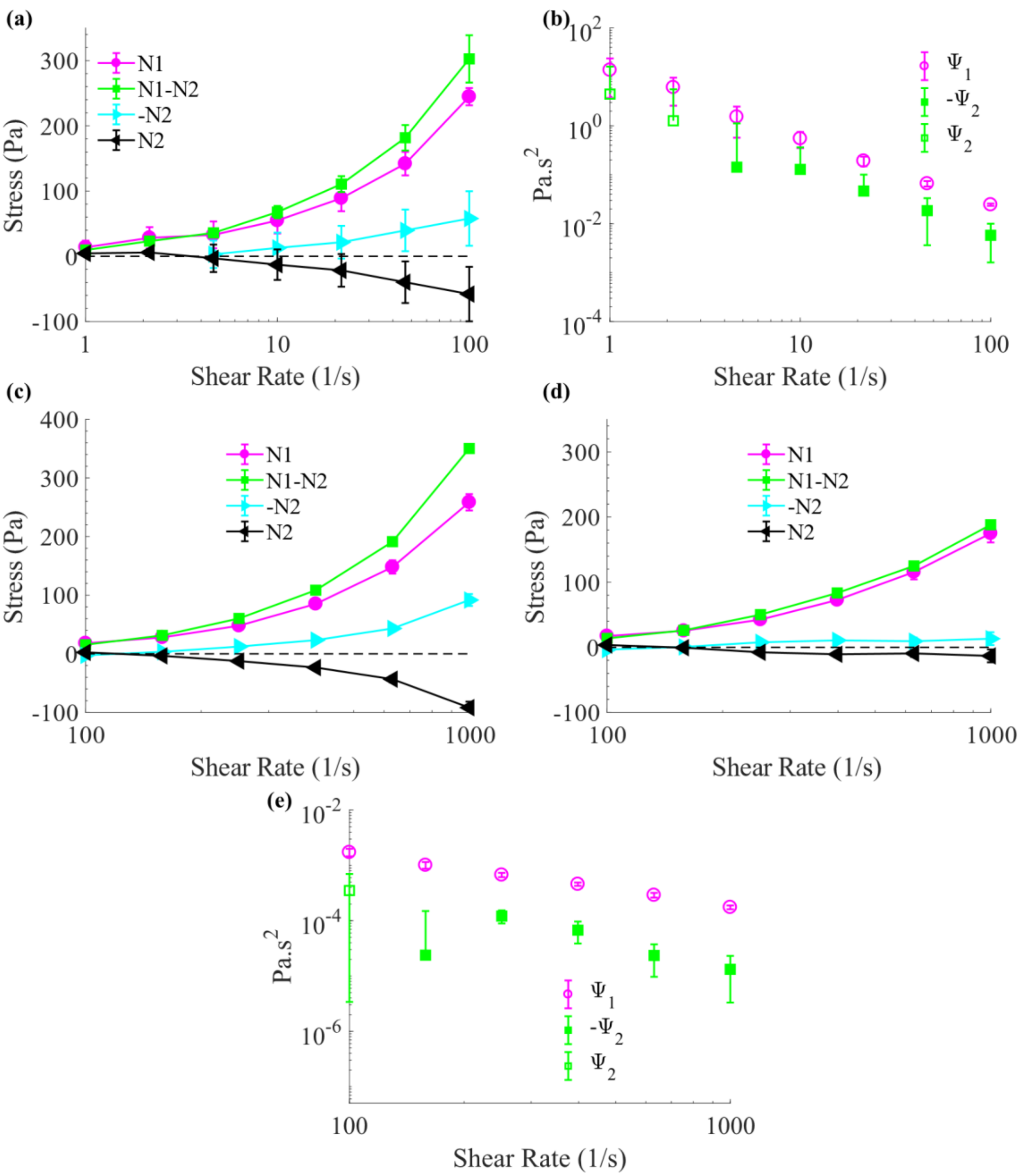

Supplementary Fig. 17. First and second normal stresses for polyacrylamide and mucin. (a) First and second normal stress differences for 2.5% polyacrylamide and respective (b) normal stress coefficients. (c) First and second normal stress differences for 10% mucin with theoretical inertial correction, with its respective normal stress coefficients shown in Fig. 5 (e). (d) First and second normal stress differences for 10% mucin with experimental inertial correction and its respective (e) normal stress coefficients. Data are presented as mean values +/- standard deviation. Statistics: (a-b) Nine independent tests were performed with cone-plate geometry and eleven tests using parallel-plate geometry; (c-e) three tests were performed for both cone-plate and parallel-plate geometries. Source data are provided as a source data file.

Supplementary Note 11: Secondary Flows from Microparticle Rotating

Here we compare the radial inward flow observed in our µPIV data for particles propelling in the z-direction [Fig. 4 (g-i)] to the secondary flows expected for nonlinearly viscoelastic fluids. The secondary flow around a rotating and translating sphere predicted by the perturbation expansion described in Supplementary Note 6 is,

$$\begin{bmatrix} v_r \\ v_\theta \\ v_\phi \end{bmatrix} = \begin{bmatrix} \frac{(-1+B_{11})(-1+r)^2(-8-3U^2+r(-4+3U^2))(1+3\cos 2\theta)}{16r^5} \\ \frac{-3(-1+B_{11})(-1+r)(-8+3(-1+r)U^2)\sin 2\theta}{16r^5} \\ \frac{3(-1+B_{11})(-1+r)U\sin 2\theta}{4r^4} \end{bmatrix}, \quad (13)$$

where r is the nondimensional radius of the sphere, U is the nondimensional velocity, θ is the cartesian angle from the sphere's propulsion axis to its center plane, and $B_{11} = \frac{b_{11}}{b_2}$ depends on the parameters of the constitutive law (see Supplementary Note 6), where each term on the left-hand side are the velocities in the spherical basis. The radial flow fields along the plane where $\theta = \frac{\pi}{2}$ were extracted from the µPIV data [Fig. 4 (g)] and plotted in Supplementary Figure 18 (a) for various angles of ϕ; the average of these is shown using the black symbols and line in Supplementary Figure 18 (a). Aside from some variation between the different ϕ values, all of the velocity profiles were markedly similar. It is important to note here that the non-zero velocity within one normalized radius of the particle is the result of the particle translating upwards during the experiment. The resulting averaged radial component was used to nonlinearly fit the parameters B_{11}, U, and the beginning and end heights of the z-slices used for averaging, by comparing the average µPIV radial flow [black curve in Supplementary Figure 18 (a)]. The best fit parameters were calculated using a delayed rejection adaptive Metropolis (DRAM) Markov chain Monte Carlo algorithm[8]. Markov chains had a length of 100,000 and only the last 10% of the chain was used to estimate the parameters. A burn-in of 10,000 was used before adaptation occurred using the DRAM process. No prior distributions were utilized but there was a restriction of $||B_{11}|| < 1$, and that the starting z-slice was limited to less than or equal to 1 radii, and the ending z-slice had to be greater than 1 radii. Additionally, the fit was normalized again by its

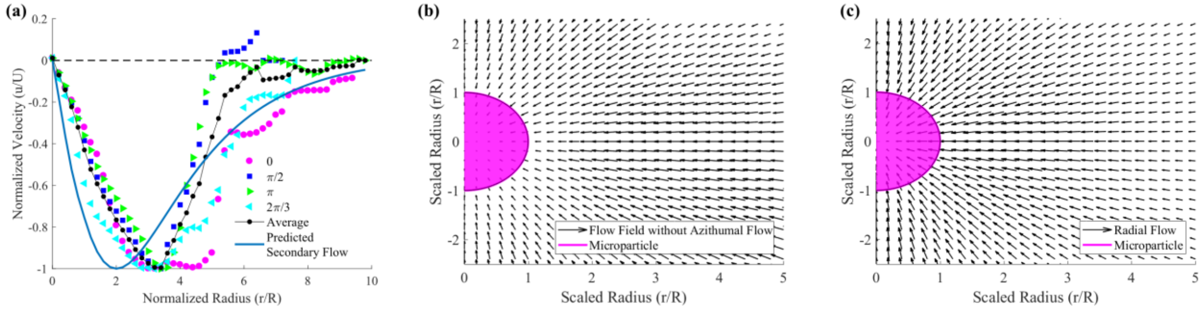

Supplementary Fig. 18. Experimental and theoretical secondary flows. (a) Experimentally extracted secondary radial flow along several angles of ϕ for $\theta = \frac{\pi}{2}$, an ensemble average between the different angles, and a fit to the ensemble average over a range of different z-planes using Supplementary Equation 13. (b) Experimentally extracted vector field of radial flow. (c) Theoretically predicted radial flow vector field using the estimated parameters and averages between the planes.

absolute maximum value to scale correctly with the ensemble averaged data. The ensemble average and the proposed fit can be found in Supplementary Figure 18 (a). The estimated values for B_{11} and U for the fit were 0.0326 and 1.2911, respectively. The optimized starting and ending z-slices were estimated to be -2.8507 and 4.2760 radii, respectively. The best-fit secondary flow prediction is similar to those of experiments, with the main difference being the radial position of the maximum magnitude of radial velocity. The vector flow field for the experimental data is shown in Supplementary Figure 18 (b) while the vector field from the estimated fit can be seen in Supplementary Figure 18 (c).

Supplementary Note 12: μPIV in 0.25% Polyacrylamide

Performing the same μPIV experiment as main text Fig. 4 (g-i) in 0.25% polyacrylamide resulted in similar flow fields to those produced in the 4% mucin experiments. These results can be seen in Supplementary Figure 19 where a 10 μm diameter microparticle was rotated at 15 Hz along the positive z-direction. The radial component of the flow field in Supplementary Figure 19 (a) matches the secondary flows predicted by the theory and is similar to the ones displayed by particles in 4% mucin; the (b) azimuthal velocity and (c) total velocity are likewise comparable.

When imaging the μPIV of a 2 μm particle, we find that the flow fields are very similar to the ones produced by the larger particles. Supplementary Figure 20 shows (a) the radial, (b) the azimuthal, and (c) total flow field for a 2 μm microparticle; unlike the 10 μm case, this particle was not in contact with the substrate and was greater than or equal to 20 μm away from it. Note that the radial inward flow remains present despite the larger distance from the substrate, indicating that the secondary flow is not caused by boundary effects. Compared to the 10 μm case, the magnitude of

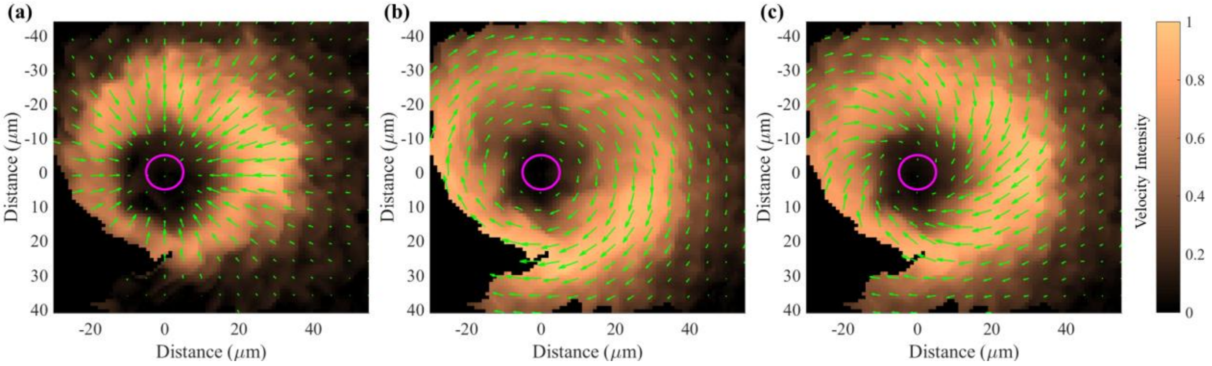

Supplementary Fig. 19. μPIV in 0.25% polyacrylamide. The (a) radial, (b) azimuthal, and (c) total velocity flow field for a microparticle propelling away from the substrate (positive z-direction). All μPIV experiments were performed close to the boundary (less than 100 μm, see Methods). Color bars to the right represents percentage of velocity magnitude with respect to the maximum velocity magnitude. These experiments were repeated over two other independent experiments.

the flows decay more rapidly as distance from the sphere increases, perhaps due to boundary effects.

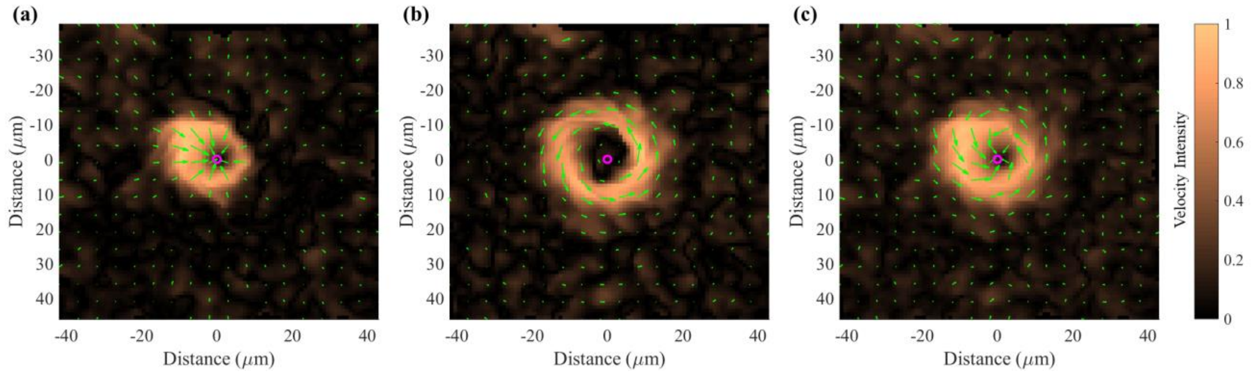

Supplementary Fig. 20. μPIV in 0.25% polyacrylamide for a 2 μm particle. The (a) radial, (b) azimuthal, and (c) total velocity flow field for a 2 μm microparticle propelling in the positive *z*-direction. The microparticle was 20 μm off the substrate. All μPIV experiments were performed close to the boundary (less than 100 μm, see Methods). Color bars to the right represents percentage of velocity magnitude with respect to the maximum velocity magnitude. These experiments were repeated over two independent experiments.

Supplementary Note 13: Additional μPIV Information

All μPIV graphs shown in the main text and SI were normalized between 0 and 1 for comparability. Provided below in Supplementary Table 1 is a listing of each figure and the maximum absolute velocities measured by the μPIV software during each experiment.

Supplementary Table 1: Maximum Velocity in μPIV Figures

Figure #	Maximum Velocity ($\mu m/s$)
Fig. 4 (a)	58.91
Fig. 4 (b)	47.21
Fig. 4 (c)	73.01
Fig. 4 (d)	59.91
Fig. 4 (e)	73.16
Fig. 4 (f)	82.26
Fig. 4 (g)	164.26
Fig. 4 (h)	162.69
Fig. 4 (i)	218.70
Supplementary Fig. 19 (a)	207.00
Supplementary Fig. 19 (b)	189.97
Supplementary Fig. 19 (c)	253.02
Supplementary Fig. 20 (a)	90.16
Supplementary Fig. 20 (b)	72.49
Supplementary Fig. 20 (c)	100.84

Supplementary References

1 Bird, R. B., Armstrong, R. C. & Hassager, O. Dynamics of polymeric liquids. Vol. 1: Fluid mechanics. (1987).

2 Giesekus, H. Die simultane Translations-und Rotationsbewegung einer Kugel in einer elastoviskosen Flüssigkeit. *Rheologica Acta* **3**, 59-71 (1963).

3 Quach, A. & Simha, R. Pressure-volume-temperature properties and transitions of amorphous polymers; polystyrene and poly (orthomethylstyrene). *Journal of Applied Physics* **42**, 4592-4606 (1971).

4 Cheang, U. K., Roy, D., Lee, J. H. & Kim, M. J. Fabrication and magnetic control of bacteria-inspired robotic microswimmers. *Applied Physics Letters* **97**, 213704 (2010).

5 Cheang, U. K., Kim, H., Milutinović, D., Choi, J. & Kim, M. J. Feedback control of an achiral robotic microswimmer. *Journal of Bionic Engineering* **14**, 245-259 (2017).

6 Ali, J., Kim, H., Cheang, U. K. & Kim, M. J. Micro-PIV measurements of flows induced by rotating microparticles near a boundary. *Microfluidics and Nanofluidics* **20**, 131 (2016).

7 Michalet, X. Mean square displacement analysis of single-particle trajectories with localization error: Brownian motion in an isotropic medium. *Physical Review E* **82**, 041914 (2010).

8 Haario, H., Laine, M., Mira, A. & Saksman, E. DRAM: efficient adaptive MCMC. *Statistics and Computing* **16**, 339-354 (2006).

9 Lai, S. K., Wang, Y.-Y., Wirtz, D. & Hanes, J. Micro-and macrorheology of mucus. *Advanced Drug Delivery Reviews* **61**, 86-100 (2009).

10 Mall-Gleissle, S. E., Gleissle, W., McKinley, G. H. & Buggisch, H. The normal stress behaviour of suspensions with viscoelastic matrix fluids. *Rheologica Acta* **41**, 61-76 (2002).

11 Alcoutlabi, M. *et al.* A comparison of three different methods for measuring both normal stress differences of viscoelastic liquids in torsional rheometers. *Rheologica Acta* **48**, 191-200 (2009).

12 Huppler, J., Ashare, E. & Holmes, J. Rheological Properties of Three Solutions. Part I. Non-Newtonian Viscosity, Normal Stresses, and Complex Viscosity. *Transactions of the Society of Rheology* **11**, 159-179 (1967).

13 Kulicke, W., Kiss, G. & Porter, R. S. Inertial normal-force corrections in rotational rheometry. *Rheologica Acta* **16**, 568-572 (1977).
