# Supplementary material for: Symmetry breaking propulsion of magnetic microspheres in nonlinearly viscoelastic fluids
Source: Nat Commun. 2021 Feb 18;12:1116. doi: 10.1038/s41467-021-21322-0 (PMC7893017; doi:10.1038/s41467-021-21322-0)
Supplement: Supplementary file 3 — Description of Additional Supplementary Files [file 41467_2021_21322_MOESM3_ESM.pdf]

Description of Additional Supplementary Files

File Name: Supplementary Movie 1
Description: The SI-Video demonstrates all major experiments discussed in the manuscript and provides examples of microparticles propelling in nonlinearly viscoelastic fluids.

File Name: Supplementary Data 1
Description: This .zip folder contains most experimental data collected and presented inside the manuscript and SI document.
